# Long-term follow-up results of endoscopic treatment of gastroesophageal reflux disease with the MUSE™ endoscopic stapling device

**DOI:** 10.1007/s00464-015-4622-y

**Published:** 2015-11-04

**Authors:** Hong Joo Kim, Chang-Il Kwon, William R. Kessler, Don J. Selzer, Gail McNulty, Amol Bapaye, Luigi Bonavina, Glen A. Lehman

**Affiliations:** Department of Internal Medicine, Kangbuk Samsung Hospital, Sungkyunkwan University School of Medicine, Seoul, Korea; Digestive Disease Center, CHA Bundang Medical Center, CHA University, Seongnam, Korea; Division of Gastroenterology and Hepatology, Department of Medicine, Indiana University School of Medicine, 550 N University Blvd, UH1634, Indianapolis, IN 46202 USA; Division of General Surgery, Department of Surgery, Indiana University School of Medicine, Indianapolis, IN USA; Shivanand Desai Center for Digestive Disorders, Deenanath Mangeshkar Hospital and Research Center, Pune, India; Division of General Surgery, University of Milan Medical School, Milan, Italy

**Keywords:** Gastroesophageal reflux disease, MUSE™, Endoscopic stapling device, GERD-HRQL, Proton pump inhibitor

## Abstract

**Background:**

The initial 6-month data for MUSE™ (Medigus, Omer, Israel) endoscopic stapling device were reported (Zacherl et al. in Surg Endosc 29:220–229, 2015). The current study aims to evaluate the long-term clinical outcome of 37 patients who received endoscopic gastroesophageal reflux disease (GERD) treatment with the MUSE™ device.

**Methods:**

Efficacy and safety data for 37 patients were analyzed at baseline, 6 months, and 4 years post-procedure. In one center (IU), efficacy and safety data were evaluated at baseline, 6 months post-procedure, and then annually up to 4 years.

**Results:**

No new complications have been reported in our long-term analysis. The proportions of patients who remained off daily PPI were 83.8 % (31/37) at 6 months and 69.4 % (25/36) at 4 years post-procedure. GERD-Health Related Quality of Life (HRQL) scores (off PPI) were significantly decreased from baseline to 6 months and 4 years post-procedure. The daily dosage of GERD medications, measured as omeprazole equivalents (mean ± SD, mg), decreased from 66.1 ± 33.2 at baseline to 10.8 ± 15.9 at 6 months and 12.8 ± 19.4 at 4 years post-procedure (*P* < 0.01).

**Conclusions:**

In our multi-center prospective study, the MUSE™ stapling device appears to be safe and effective in improving symptom scores as well as reducing PPI use in patients with GERD. These results appeared to be equal to or better than those of the other devices for endoluminal GERD therapy. Future studies with larger patient series, sham control group, and greater number of staples are awaited.

The aims of treatment in gastroesophageal reflux disease (GERD) include relief of symptoms, healing of esophagitis if present, prevention of symptom recurrence, and prevention of complications such as esophageal ulcers, peptic strictures, and Barrett’s esophagus. Both medical therapy with antacids or proton pump inhibitors (PPI) and surgical treatments decrease symptoms and improve the quality of life in GERD [1–3]. Currently, acid suppression with PPI therapy remains the most widely used treatment option, being highly effective in symptom relief, as well as in healing and maintaining remission [4–6]. Pharmacologic therapy, however, often requires long-term treatment, and some patients are unwilling to take daily medication for prolonged periods of time or even for lifelong. Additionally, up to 40 % of patients do not have a complete response to PPI treatment [7–10]. Surgical treatment, such as fundoplication, is indicated when the above measures fail or at the patient’s request. While laparoscopic fundoplication is the treatment of choice for the surgical treatment of GERD, its inherent invasive nature as a surgical procedure remains. As alternatives to drugs and surgery, a number of endoscopic techniques have been developed to treat GERD [11, 12]. The purpose of these endoscopic procedures is to modify the gastroesophageal junction (GEJ) to decrease reflux from the stomach into the esophagus. Initial studies demonstrated promising clinical results and short-term efficacies of the new endoscopic therapies; however, the long-term follow-up results are less often reported [11, 12].

Recently, a short-term follow-up result of endoscopic anterior fundoplication using a novel transoral endoscopic device (MUSE™, formerly called SRS; Medigus, Omer, Israel) has been published and shown to be generally safe and effective as an alternative endoscopic GERD therapy [13]. In that report, safety and efficacy data for MUSE™ stapling device obtained from six international sites were compared at baseline and 6 months post-procedure. The GERD-Health Related Quality of Life (HRQL) scores (off PPI) improved by >50 % in 73 % (48/66) of patients, and 42 patients (64.6 %) were no longer using daily PPI medication. The mean percent of total time with esophageal pH ≤ 4.0 decreased from baseline to 6 months post-procedure. Two severe adverse events (SAEs) requiring intervention were reported in this study. This multi-center prospective clinical study evaluated the long-term safety and efficacy of endoscopic treatment with the MUSE™ endoscopic stapling device which was used to treat 37 patients with GERD. Three of the initial six centers followed their patients for 4 years and are the subjects of this report.

## Patients and methods

The current long-term follow-up data are an extension to the previously mentioned initial 6-month data report of the multi-center prospective clinical trial of the MUSE™ endoscopic stapling device (identifier: NCT00734747) [13]. Three international centers (one in Europe, one in India and one in the USA) participated in this long-term follow-up trial, with each site obtaining institutional review board approval. Informed consent was obtained from all participants.

### Patients

This prospective multi-center clinical trial included patients aged 18–70 years with ≥2 years of documented GERD symptoms and ≥6 months of continuous PPI therapy treated between May 2008 and November 2010. Full details of the inclusion and exclusion criteria for the enrollment of our patients were described in a previously published report [13]. In one center (Indiana University, abbreviated as IU), efficacy and safety data were evaluated at baseline, 6 months post-procedure, and then annually for 4 years. Procedure safety was determined by evaluation of all treatment-related adverse events. All data were obtained by phone interview, mail survey, or direct patient contacts at clinic.

### Device and procedure

The full descriptions for the composition of the device (the MUSE™ endostapler, designed to be operated by a single user) and the endostapling procedure were detailed in a previously published report [13]. The incident deserving special mention was the protocol amendment after the 24 cases to reduce the pressure gradient between the abdominal and thoracic cavity in order to prevent air leaks around the anchoring screws. Initially, positive end-expiratory pressure (PEEP) of 5 mmHg (6.8 cm H_2_O) was applied only to patients with a sliding hiatal hernia (SHH, patients with SHH ≥ 3 cm were excluded from the initial enrollment). After the amendment, all subjects were ventilated with a PEEP setting of 5 mmHg, after the orotracheal intubation, and if SHH were still evident after the application of 5 mmHg PEEP, PEEP was gradually increased to 10 mmHg until the hernia was reduced. The proportion of patients with small hiatal hernia <3 cm was 21.6 % (eight of 37 enrolled patients had reducible small hiatal hernia). In the first 24 subjects, two serious adverse events (SAEs) occurred, including a case of empyema and pneumothorax due to esophageal leak and a case of upper gastrointestinal hemorrhage. For mitigation of risk, the procedure protocol was amended to apply additional stapling (maximum three sets of 5 each) and to require prophylactic anti-emesis treatment to prevent immediate post-operative retching with the aim of reducing stress at the stapling site. The protocol was also amended to require a chest X-ray to confirm no leaks are present prior to hospital discharge. In addition, device changes were made to prevent air insufflation during screw insertion in order to prevent the tendency of air to leak into the peritoneum around the screws before the staples are formed. Following these amendments and protocol changes, there were no further cases of leak or pneumoperitoneum.

### Assessment of efficacy and safety

Enrolled patients completed the GERD-HRQL questionnaire and a medication list indicating the names, dose, and the frequency of the anti-secretory drugs at the time points of baseline, 6 months, 1, 2, 3, and 4 years post-procedure. The GERD-HRQL measures were administered twice during the pre-procedure phase: once while on PPI medication and again after having discontinued the medication for 7 days. An upper gastrointestinal endoscopy was performed at baseline to evaluate the presence and size of hiatal hernia and the grade of esophagitis in correspondence to established study inclusion criteria. Esophageal pH monitoring was measured and manometry performed at baseline with patients off anti-secretory medications for at least 7 days. At month 6, patients underwent repeat esophageal pH measurement, esophageal manometry, and standard upper gastrointestinal endoscopy, after the patients were off PPI therapy for a minimum of 7 days. Esophageal pH measurement and manometry were not repeated after the 6 months post-procedure. Adverse events were evaluated at each visit of time 0, weeks 1, 4, 12, month 6, and years 1, 2, 3, and 4 as well as at any unscheduled visits. SAEs were defined as those that resulted in death, were life-threatening, or required prolongation of a current hospitalization. Per protocol, hospitalization was allowed for up to 72 h following the procedure. Hospitalization days beyond this period were recorded as a SAE.

### Statistical analysis

The safety measures and reduction in GERD-HRQL score in total patients cohort (multi-center trial including 37 patients) and patients cohort of IU (*n* = 21) at the follow-up time points were analyzed as a primary end point in this study. The proportions of patients who were off daily PPI medications and daily dosage measured as omeprazole equivalents (mg) in total patients cohort and patients cohort of IU at the follow-up time points were analyzed as secondary end points in this study. Due to the nonparametric distribution of most of the continuous data, comparisons between baseline and post-procedure results (reduction in GERD-HRQL score, daily dosage of GERD medications measured as omeprazole equivalents, total time distal esophageal pH ≤ 4.0, and DeMeester score) were tested by Wilcoxon signed-rank test (2-related samples) or Kendall’s *W* test (K-related samples) where appropriate, at a *P* value of 0.05. Analyses of dichotomous variables (proportions of patients who were off daily PPI medication) were performed using Fisher’s exact test. Statistical analysis was conducted using the SPSS version 13.0 software package (SPSS Inc., Chicago, IL).

## Results

Initially, 64 patients who consented and enrolled in the initial short-term follow-up study [13] across 6 international sites served as the population for this long-term safety and efficacy analysis. Four-year data were collected from only three of the six centers with 39 total patients. Of these, 37 patients had full 4-year follow-up data (37/39). The baseline characteristics of the total patients cohort and IU cohort are outlined in Table 1.

**Table 1:** Baseline characteristics

Characteristics	Total patients cohort (*n* = 37)	IU cohort (*n* = 21)	*P* value
Age (mean ± SD, years)	44.7 ± 13.3	48.4 ± 14.7	NS
Male gender (%)	20 (54.1)	11 (52.4)	NS
BMI (mean ± SD, kg/m^2^)	26.2 ± 4.9	28.6 ± 3.9	NS
GERD-HRQL score (mean ± SD, off PPI)	29.1 ± 5.6	30.9 ± 6.3	NS
GERD-HRQL score (mean ± SD, on PPI)	13.3 ± 6.4	13.6 ± 6.3	NS
Daily PPI use (%)	37 (100.0)	21 (100.0)	NS
Daily dosage of GERD medications, measured as omeprazole equivalents (mean ± SD, mg)	66.1 ± 33.2	80.2 ± 31.4	NS
% Time pH < 4.0 (mean ± SD)	12.7 ± 13.2	11.2 ± 6.9	NS
DeMeester score (mean ± SD)	49.4 ± 47.2	41.1 ± 22.7	NS

*IU* Indiana University, *SD* standard deviation, *NS* not significant, *BMI* body mass index, *kg* kilogram, *GERD-HRQL* Gastroesophageal Reflux Disease-Health Related Quality of Life, *PPI* proton pump inhibitor

### Primary outcomes

**Fig. 1:**
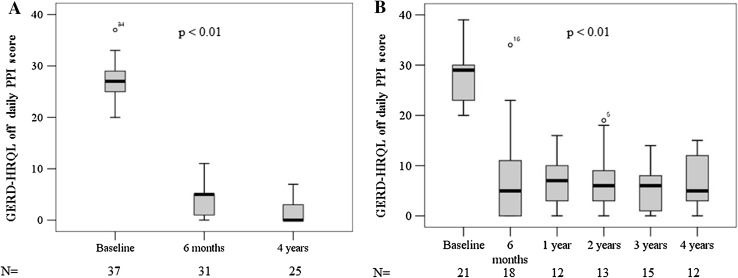
The changes in GERD-HRQL off daily PPI scores in **A** multi-center trial including three sites and **B** subset of one center (IU) with annual follow-up data

After 6 months of follow-up, no new SAEs were reported in our long-term analysis. The SAEs occurred during the initial 6-month follow-up were previously reported [13]. Improvement in GERD symptoms, as measured by the reduction in GERD-HRQL score in our total patients group, was accomplished during the follow-up period of 4 years. The mean ± SD GERD-HRQL scores (off PPI) of the total patients group improved from 29.1 ± 5.6 to 8.9 ± 8.3 at 6 months (*P* < 0.01, compared to baseline) and 5.3 ± 5.8 at 4 years post-procedure (*P* < 0.01, compared to baseline and 6 months, Fig. 1A). Annual follow-up data obtained in one center (IU) revealed similar data for GERD-HRQL scores (mean ± SD, off PPI) compared to those of total patients group (Fig. 1B).

### Secondary outcomes

**Fig. 2:**
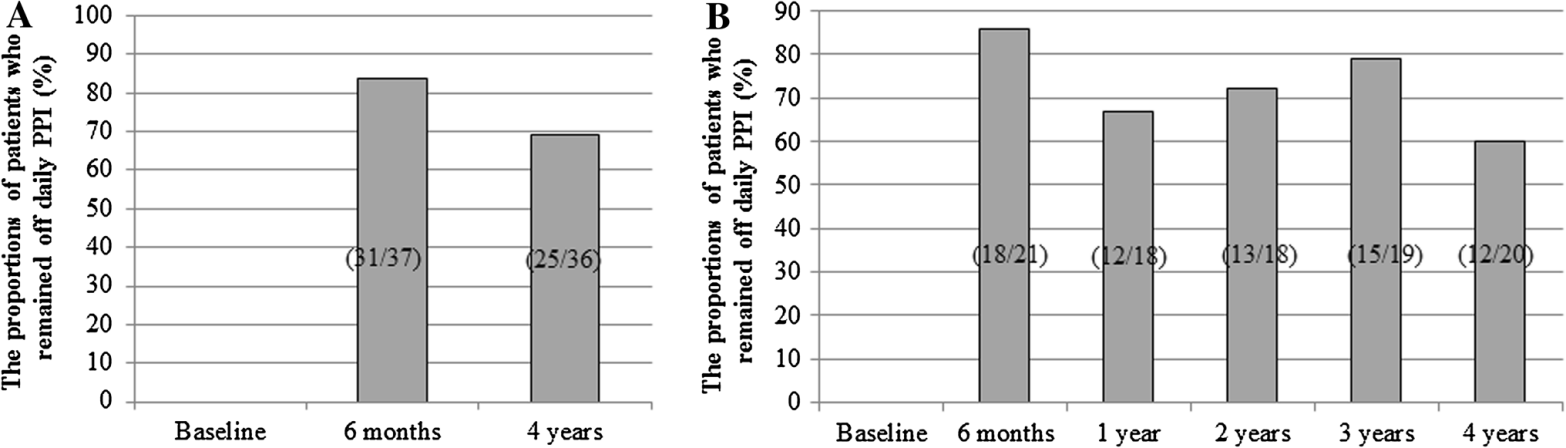
The proportions of patients who remained off daily PPI in **A** multi-center trial including three sites and **B** subset of one center (IU) with annual follow-up data

**Fig. 3:**
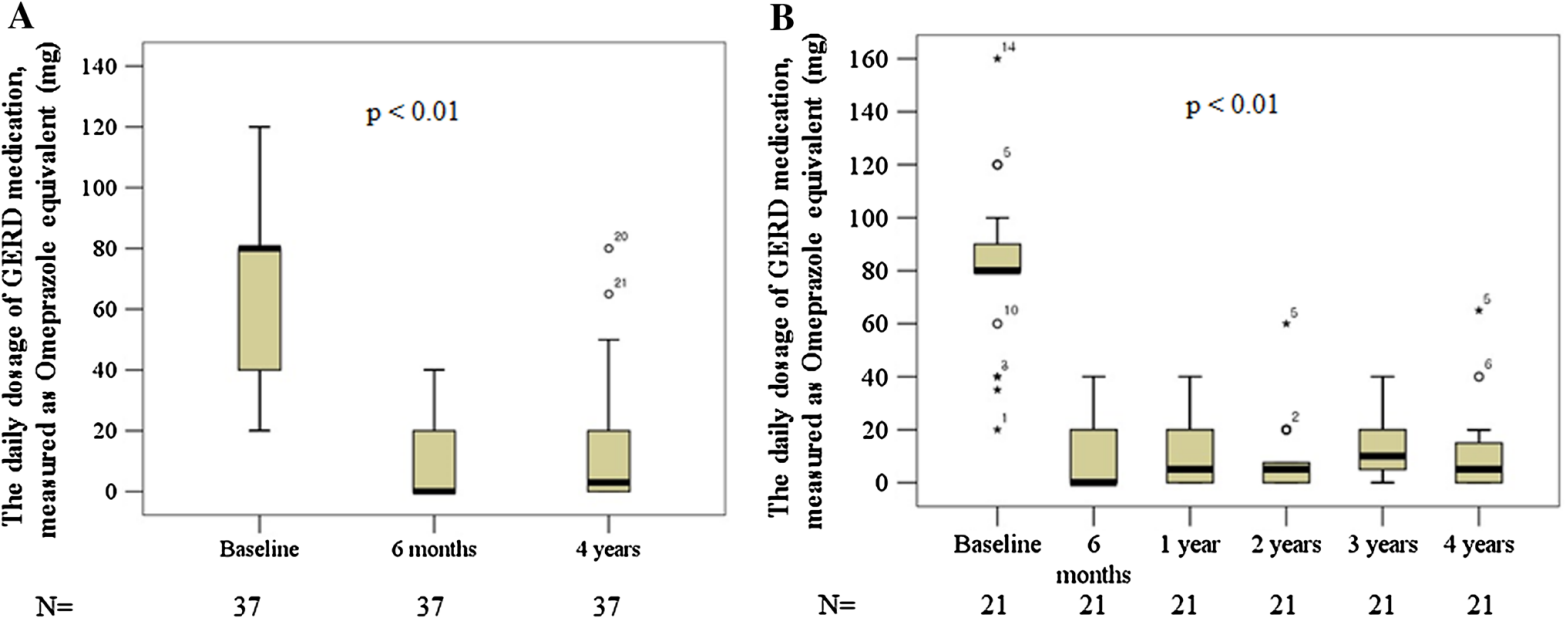
The daily dosage of GERD medication, measured as omeprazole equivalents (mg) in **A** multi-center trial including three sites and **B** subset of one center (IU) with annual follow-up data

The proportions of patients who remained off daily PPI medication in the total patients group were 83.8 and 69.4 % at 6 months and 4 years post-procedure, respectively (Fig. 2A). The proportions of patients who remained off daily PPI medication in the IU group were similar to those of total patients group (Fig. 2B). The daily dosage of GERD medications, measured as omeprazole equivalents (mean ± SD, mg), for the total patients group decreased from 66.1 ± 33.2 at baseline to 10.8 ± 15.9 and 12.8 ± 19.4 at 6 months and 4 years post-procedure, respectively (*P* < 0.01, compared to baseline, Fig. 3A). The daily dosage of GERD medications (mean ± SD, mg) for IU group was also decreased similar to those for total patients group (Fig. 3B). Although the patients remaining on daily acid suppression therapy after 4 years of MUSE treatment were still substantial, they had lower symptom scores, and most had reduced dose of PPI medication. No patient required laparoscopic Nissen fundoplication.

### Improvement in acid exposure

**Fig. 4:**
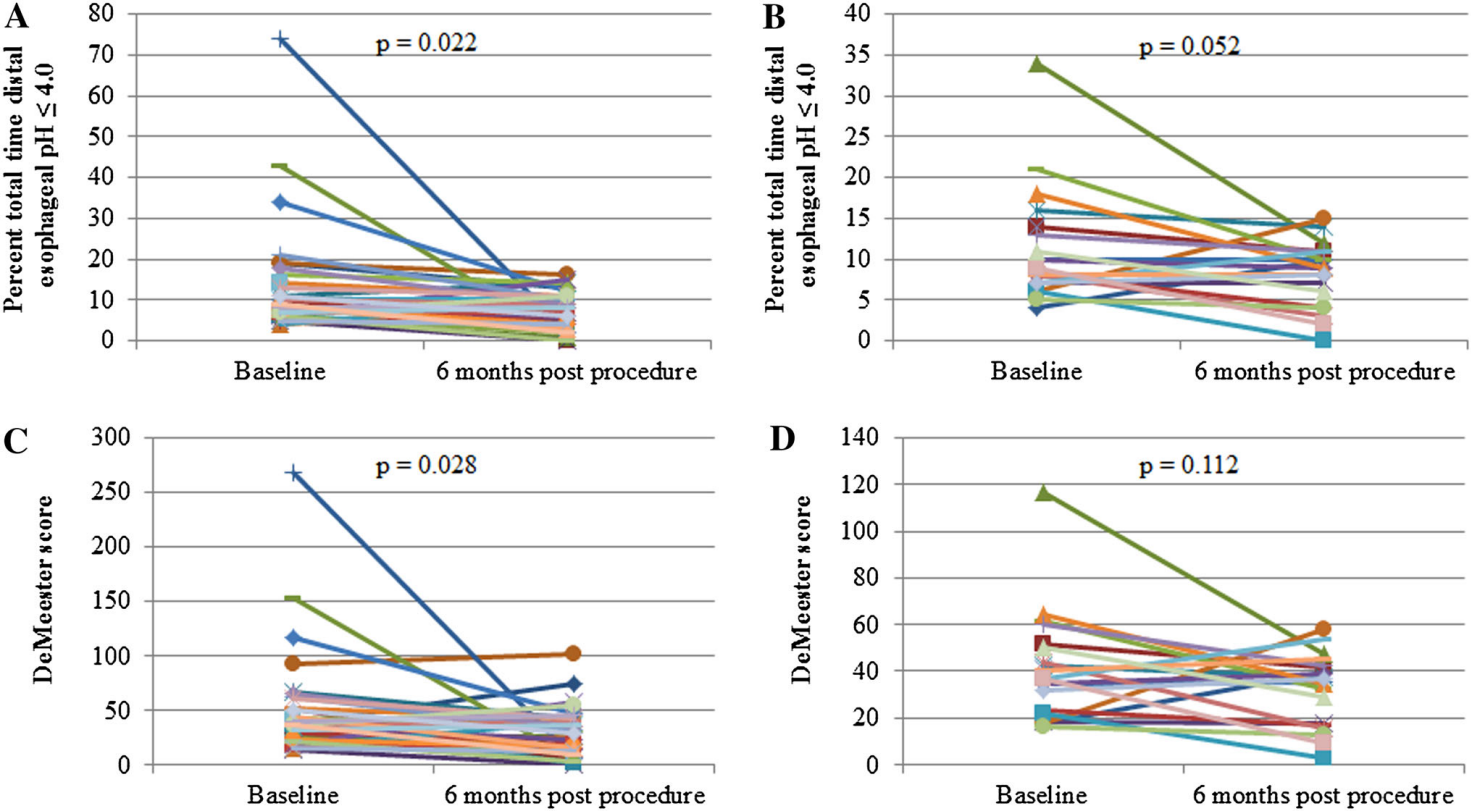
Both total patients group and IU subset group showed a reduction in acid reflux, as measured by wireless 48-h pH monitoring administered at baseline and 6 months post-procedure. Percent total time pH ≤ 4.0 (mean ± SD) was decreased from baseline to 6 months post-procedure in **A** total patients group (12.7 ± 13.2 to 7.0 ± 4.7) and **B** IU subset group (11.2 ± 6.9 to 8.2 ± 4.0). DeMeester scores (mean ± SD) were also decreased from baseline to 6 months post-procedure in **C** total patients group (49.4 ± 47.2 to 29.1 ± 22.0) and **D** IU subset group (41.1 ± 22.7 to 32.5 ± 15.2)

According to the initial short-term follow-up study, pathologic gastroesophageal acid reflux was defined as >4.5 % total time with pH < 4.0 or a DeMeester composite score >14.7. In the current study, 13 (37.1 %) of 35 enrolled patients (two subjects did not complete the 6-month pH study) showed normalization of their 6-month pH study by 24- or 48-h ambulatory pH study results. Both total patients group and IU group experienced a reduction in acid reflux, as measured by intraesophageal pH monitoring administered at baseline and 6 months post-procedure. The mean ± SD percent total time distal esophageal pH ≤ 4.0 improved from 12.7 ± 13.2 % at baseline to 7.0 ± 4.7 % at 6 months post-procedure in total patients group (44.9 % reduction, *P* = 0.022, Fig. 4A). The mean ± SD DeMeester score of the total patients group improved from 49.4 ± 47.2 to 29.1 ± 22.0 at 6 months post-procedure (41.1 % reduction, *P* = 0.028, Fig. 4C). The mean ± SD percent total time distal esophageal pH ≤ 4.0 (Fig. 4B) and the mean ± SD DeMeester score (Fig. 4D) in the IU group also improved from baseline to 6 months post-procedure. However, it did not reach statistical significance unlike the total patients group.

## Discussion

In our multi-center prospective study, the MUSE™ endoscopic stapling device was shown to be relatively safe and efficacious with more than 4 years of follow-up for patients with PPI-responsive, moderate-to-severe GERD. Our primary study end points were safety profiles and the comparisons of effectiveness of MUSE™ endoscopic stapling device measured as reduction in GERD-HRQL score. Most SAEs were reported in the immediate post-procedural period and were concentrated in the first 24 subjects. The introduction of protocol amendments as the use of anti-retching prophylaxis, increased number of staplings, and air insufflation control during the screw deployment resulted in much improved safety profile in the remaining subjects enrolled, and no additional cases of leakage or pneumomediastinum were reported. Additionally, no new residual SAEs have been reported in our long-term follow-up after 6 months of this procedure.

Reduction in GERD-HRQL score by the range of 69–82 % at each time point of follow-up is comparable to those achieved in other endoscopic therapies for GERD [11, 12, 14–16]. A reduction in GERD-HRQL scores for the NDO Plicator (NDO Surgical, Inc., Mansfield, MA, USA) from baseline to 36 months was reported to be 19 to 8 [14] and similar or slightly inferior to those (29.1 to 5.3 in our total patients group, and 30.9 to 7.3 in IU group) seen in the current study. When compared to transoral incisionless fundoplication (TIF 2.0) using EsophyX™ device (EndoGastric Solutions, Redmond, WA, USA), patients in our study had lower GERD-HRQL scores at baseline (29.1 vs. 46) with similar improvements at 6-month follow-up (8.9 vs. 15) [15]. Ten-year follow-up data for radiofrequency modulation of lower esophageal sphincter or Stretta^®^ procedure [16] showed significant decrease in GERD-HRQL scores (baseline 27.81 to 8.55 at 10-year follow-up). Although only 4-year follow-up data were available in the current study, decrease in the mean GERD-HRQL score (off PPI, 29.1 to 5.3 at 4 years) showed similar results to those of long-term follow-up study for Stretta^®^ procedure [16]. Sustained reduction in GERD-HRQL score up to 4 years was seen in our study. So far, there have been only a few reports concerning the long-term durability of therapeutic effectiveness for endoscopic treatment of GERD [14–16].

The secondary end points of the current study were the proportions of patients who were off daily PPI medications and daily dosage of GERD medications measured as omeprazole equivalents (mg) in total patients cohort and IU cohort at the follow-up time points. Increases in the proportion of patients off daily PPI medication were observed in both total patients group and IU group up to 4 years after the treatments (Fig. 2). Long-term follow-up data for NDO Plicator^14^ showed similar results to the current study for the proportion of patients off daily PPI medication and revealed that 57 % (16/28) of baseline PPI-dependent patients remained off daily PPI therapy. For the EsophyX™ device [15], mid-term (2 years) proportion of patients off daily PPI (69.2 % in EsophyX™ vs. 72.2 % in the IU group of the current study) also revealed similar results to the current study. Ten-year follow-up data for Stretta^®^ procedure [16] showed that 50 % or greater reduction in PPI use compared to baseline was achieved in 64 % of patients and in 41 %, PPIs were entirely eliminated.

A reduction in daily dosage of GERD medications, measured as omeprazole equivalents (mean ± SD, mg) up to 4 years after the procedure, further supports durable symptomatic improvement (Fig. 3). The similar decreases in daily dosage of GERD medications were observed in the studies using other endoscopic plication devices as well [14–16].

The 44.9 % reduction in the mean total time distal esophageal pH ≤ 4.0 in total patients group at 6 months after the procedure was seen (*P* = 0.52 vs. baseline). This trend was superior to data (15.8 %) reported in NDO Plicator study. The 41.1 % reduction in the mean DeMeester score in total patients group at 6 months after the procedure was also superior to data (15.9 %) reported in NDO Plicator study [14]. The pH monitoring data obtained 6 months after transoral incisionless fundoplication (TIF 2.0) with EsophyX™ also showed similar results to our study [15]. The percentage of reflux episodes reaching the proximal esophagus tended to be lower, but there was no difference in the number of weakly alkaline refluxes. The number of weakly acidic refluxes decreased after treatment, though not significantly. The DeMeester score also did not change. The LES pressure and distal esophageal amplitude did not change after treatment.

Important limitations in the design of the current study include a small number of enrolled patients and the lack of a sham or control group. GERD has been shown to have a placebo response rate of at least 25 percent, as shown by results from sham control studies [17, 18]. Subjective improvements in outcomes such as symptoms and QOL may not necessarily correlate with objective measurements such as gastroesophageal acid reflux, and controversies continue over the mode of action of various endoscopic therapies.

In conclusion, the current multi-center prospective study reports the long-term follow-up results of safety and therapeutic effectiveness of MUSE™ endoscopic stapling device in patients with GERD. MUSE™ endoscopic stapling device appears to be safe and effective in improving symptom scores as well as reducing PPI use in patients with GERD. Further studies with longer-term follow-up results of intraesophageal pH monitoring and with a sham control group are awaited.
